# Fatigue-life and stress distribution of a glass-ceramic under different loading conditions

**DOI:** 10.1590/0103-6440202305129

**Published:** 2023-03-06

**Authors:** Tábata Mariana da Silva Dalla Lana, Kátia Raquel Weber, Juliana Arisi Medeiros, Fábio Goedel, Paula Benetti, Márcia Borba

**Affiliations:** 1 Graduate Program in Dentistry, Dental School, University of Passo Fundo(UPF), Passo Fundo, Rio Grande do Sul, Brazil.; 2 Engineering School, University of Passo Fundo(UPF), Passo Fundo, Rio Grande do Sul, Brazil.

**Keywords:** ceramics, biomechanics, stress, fatigue

## Abstract

This study aimed to evaluate the effect of different loading conditions on the mechanical behavior and stress distribution of a leucite-reinforced glass-ceramic. Plate-shaped ceramic specimens were obtained from leucite-reinforced glass-ceramic (1.5 × 8.4 × 8.3 mm) and adhesively cemented to a dentin analog substrate. Monotonic and cyclic contact fatigue tests were performed to simulate sphere-to-flat contact, using a 6 mm diameter spherical piston; and flat-to-flat contact, using a 3 mm diameter flat piston. For the monotonic test (n=20), a gradual compressive load (0.5 mm/min) was applied to the specimen using a universal testing machine. Failure load data were analyzed with Weibull statistics. The cyclic contact fatigue test was performed using protocols (load and a number of cycles) defined by the boundary technique (n=30). Fatigue data were analyzed using an inverse power law relationship and Weibull-lifetime distribution. The stress distribution was investigated using Finite Element Analysis (FEA). The monotonic and the fatigue Weibull modulus were similar among the two contact conditions. In fatigue, the slow crack growth exponent was greater for sphere-to-flat contact, which indicates that the load level had a greater effect on the specimen’s probability of failure. In conclusion, FEA showed different stress distribution for the tested loading conditions. The stress distribution and probability of fatigue failure of specimens tested in sphere-to-flat contact showed greater dependency to load level.

## Introduction

Clinical studies report a 5-year survival rate of 96.6% for glass-ceramic crowns ^1^. Nevertheless, technical complications related to the mechanical stability of the ceramic materials are important sources of failure, resulting in catastrophic and minor fractures (i.e. chipping) ^1^ ^-^ ^5^. To predict the mechanical behavior of ceramic restorations and prevent clinical failures, data from laboratory studies are frequently used. However, there are several limitations in these studies, mainly related to the high amplitude of the load applied in the restorations, ^3^ ^,^ ^6^ ^,^ ^7^ the type of piston used to apply the load ^8^ ^-^ ^11^, and the type of supporting substrate ^12^ ^,^ ^13^.

Most studies have used spherical pistons of small diameter against flat ceramic surfaces, creating sphere-to-flat contacts. The diameter of these pistons varies between 4 and 6 mm, aiming to simulate the diameter of a tooth cusp ^3^ ^-^ ^6^ ^,^ ^8^ ^,^ ^10^ ^,^ ^14^ ^,^ ^18^ ^,^ ^19^. Due to the small area in which the load is distributed, a high pressure is induced in the contact surface, which may lead to the development of cone cracks and does not accurately represent the clinical conditions ^8^. Experiments using sphere-to-flat contacts showed that contact damage could be minimized by increasing the piston radius, justifying the recent studies that use 40-mm diameter hemispherical pistons ^3^ ^,^ ^6^ ^,^ ^10^. Additionally, clinically, contact facets are developed between the opposing teeth, with dimensions varying from 0.5 to 3.0 mm in diameter to maintain the contact pressure up to 40 MPa ^6^. Based on these assumptions, Kelly ^3^, suggested the use of a flat-tip piston with a contact area of 2 to 3 mm in diameter to create flat-to-flat contacts as the ones observed clinically. This type of piston could simulate the stress distribution that ceramic restorations are subjected intra-orally and induce failure modes found clinically for glass-ceramics restorations^8^. Nevertheless, more information is needed on how to design mechanical tests using flat-to-flat contact conditions, as several variables are involved in the experiments ^11^.

Mechanical tests can be performed using hard (metal and ceramic) and soft (composite) pistons ^5^ ^-^ ^11^ ^,^ ^13^ ^-^ ^19^. Although soft materials have similar elastic properties than the tooth; metal, in a sphere-to-flat contact condition, is still the most commonly used loading piston ^5^ ^,^ ^8^ ^,^ ^10^ ^,^ ^11^ ^,^ ^14^ ^,^ ^17^ ^-^ ^19^. Variables involved in the contact damage of brittle materials has been extensively investigated ^4^ ^,^ ^5^ ^,^ ^8^ ^,^ ^10^ ^,^ ^14^ ^,^ ^17^. Yet, characterizing these variables using a less-controlled oral environment simulation can be a challenge. In fatigue, ceramics fail under lower stress levels than those found in fast fracture tests and show different failure modes, being a more clinically relevant methodology ^5^ ^,^ ^7^ ^,^ ^15^ ^-^ ^17^. Furthermore, understanding the failure behavior of ceramics under different testing conditions allows the researchers to choose the parameters that most closely simulate the clinical behavior ^3^ ^,^ ^6^.

Therefore, the aim of this in vitro study was to investigate the effect of different loading conditions on the failure behavior and stress distribution of a leucite-reinforced glass-ceramic bonded to a dentin analog substrate. Sphere-to-flat and flat-to-flat contact conditions, using a stainless-steel metallic piston, were investigated in monotonic compressive load and cyclic fatigue setups. The 6-mm diameter stainless steel spherical piston was chosen to represent sphere-to-flat conditions as it was the most frequently used in laboratory studies ^4^ ^,^ ^5^ ^,^ ^10^ ^,^ ^14^ ^,^ ^18^ ^,^ ^19^. The research hypothesis was that the loading conditions affect the mechanical behavior and stress distribution of glass-ceramic specimens.

## Material and methods

Plate-shaped specimens of a leucite-reinforced glass ceramic (VL, IPS Empress CAD; Ivoclar Vivadent, Schaan, Liechtenstein) were produced with the dimensions of 1.5 × 8.4 × 8.3 mm. Prefabricated and fully sintered CAD-CAM blocks were cut in slices with a diamond disk in a metallographic cutting machine (Miniton model; Struers, Copenhagen, Denmark), under water cooling. Ceramic slices were flattened using 180-, 280-, and 500-grit papers and polished with 600- and 1000-grit papers under constant water irrigation to obtain the final thickness of 1.5 mm. A material analogous to dentin (glass-fiber reinforced epoxy resin, NEMA G10; International Paper, Hampton, NY, USA) was used as supporting substrate (4 mm thick × 20 mm diameter) ^3^ ^,^ ^6^ ^,^ ^13^ ^,^ ^20^.

For the cementation protocol, ceramic and G10 substrate surfaces were etched using 10% hydrofluoric acid (HF) for 60 seconds (Condac Porcelana; FGM, Joinville, Santa Catarina, Brazil), washed with water for 30 seconds and air-dried for 30 seconds ^7^ ^,^ ^18^. A silane bonding agent (Prosil; FGM, Joinville, Brazil) was applied and left to evaporate for 60 seconds. G10 substrate was etched with HF to expose the glass fibers and create micro-mechanical retentions in order to improve the bond strength ^7^ ^,^ ^9^ ^,^ ^18^ ^,^ ^20^.

Ceramic specimens were cemented onto the G10 substrate with a dual-polymerized self-adhesive resin cement (Rely X U200; 3M Dental Products, Sumare, São Paulo, Brazil) following the manufacturer’s instructions. A cementation device was used to apply a constant load (7.35 N for 3 minutes) on the ceramic specimen during the cementation procedure aiming to obtain a uniform cement layer ^7^ ^,^ ^9^. Specimens were stored in distilled water at 37 °C for 48 hours prior to the mechanical tests. A total of 100 specimens were produced.

Specimens were evaluated with monotonic compressive load and cyclic contact fatigue tests using stainless steel pistons to simulate sphere-to-flat contact (SFC), using a 6 mm diameter spherical piston; and flat-to-flat contact (FFC), using a piston with a 3 mm diameter flat contact area.

For the monotonic test, a gradual compressive load was applied to the ceramic surface using a universal testing machine (DL 2000; EMIC, Sao Jose dos Pinhais, São Paulo, Brazil), at a crosshead speed of 0.5 mm/min, in 37 °C distilled water. During load application, acoustic monitoring was performed, and the test was interrupted at the sound of the first crack (sharp sound wave)^7^ ^,^ ^9^. Forty specimens were evaluated, half in each contact condition (n=20).

The fracture load data (L_f_ in N) were analyzed with two-parameter Weibull analysis to obtain the characteristic fracture load (L_0_) and the Weibull modulus (*m*) ^21^. The 95% confidence intervals (95% CI) of *m* and L_0_ were calculated using tabulated values ^21^.

Cyclic contact fatigue was performed using a pneumatic mechanical cycling machine (Biocycle; Biopdi, Sao Carlos, São Paulo, Brazil) with 2 Hz (*R*=0), in distilled water at 37 °C. The piston was always in contact with the ceramic surface during the test in order to avoid impact. Sixty specimens were tested in fatigue and were evenly distributed into the 2 contact conditions (n=30). For each contact condition, 2 lifetimes were used (100,000 and 200,000 cycles), each one with 2 constant load levels (L_1_ and L_2_), defined according to the boundary technique ^7^ ^,^ ^15^ ^,^ ^22^ ^-^ ^24^.

For SFC, the first 10 specimens were tested at 98 N load (L_1_) for 100,000 cycles, based on data from a previous study ^7^. All specimens failed by the end of the protocol. Then, a second load level (L_2_) was calculated according to Equation 1 ^22^ ^,^ ^23^:

L2=L1+S.1-in. L1→ i<0.5nL1- S. in .L1            → i ≥0.5n Equation 1

Where *i* is the number of specimens that failed up to the preset number of cycles in L_1_, n is the total number of specimens tested in L_1_ (n=10), and *S* is a constant (0.178) selected to minimize the chance of all or none of the specimens failing at L_2_.

A new set of 10 specimens, for SFC, were tested with 81 N (L_2_) and 80% failed up to 100,000 cycles. The specimens that survived the test were allowed to run out through the second lifetime of 200,000 cycles (L_2_ for 100,000 cycles was used as L_1_ for 200,000 cycles). No specimens failed, so the failure rate remained at 80%. Based on these data, the second load level for the second lifetime was calculated, resulting in 69 N (L_2_). Ten new specimens were cycled and 60% failed between 0 and 200,000 cycles.

For FFC, the load level (L1) chosen to test the first 10 specimens for 100,000 cycles was 69 N. A lower load was chosen based on data from a pilot study and considering the low survival rate of specimens tested with 98 N and 81 N in SFC. All specimens failed after 100,000 cycles. Then, a new set of 10 specimens were cycled at 56 N (L_2_) for 100,000 cycles, and 70% failed by the end of the protocol. The surviving specimens were allowed to run out through the second lifetime of 200,000 cycles and no specimens failed. The second load level for 200,000 cycles was calculated, being 49 N (L_2_). Ten new specimens were tested and 70% failed by the end of the protocol.

Specimen failure was considered when cracking, chipping or catastrophic fracture occurred, after the preset number of cycles. Failed specimens were analyzed using transillumination with blue light and an optical microscope. Cracks were classified as: ^1^ radial crack, located on the intaglio surface of the ceramic, at the cementation interface; ^2^ cone crack, located on the ceramic surface in contact with the piston; ^3^ combined, when both types of cracks were present ^7^ ^,^ ^9^ ^,^ ^16^ ^,^ ^19^.

Fatigue data were analyzed with an inverse power law lifetime-stress relation (IPL) and a Weibull lifetime distribution (MLE - Maximum Likelihood Estimation), using a statistical software (ALTA 11; Reliasoft). This model considers both failure and survival data (censored). Different loads and lifetimes were used in the fatigue test (following the boundary technique) to obtain a low and high probability of failure data, which could increase the power of the mathematical model used to predict the fatigue behavior of the experimental groups at different scenarios ^24^. Probability of failure (P_f_) predictions with respective 95% confidence intervals for 30 N to 80 N loads were made based on the statistical model. The combined IPL-Weibull model was (Eq. 2):

$$P_{f}=1-exp\left(K\sigma^{n}t\right)1/\beta$$ Equation 2

where P_f_ is the probability of failure at time *t*, α is the stress level, ( is the fatigue Weibull modulus, *n* is the exponent of crack growth, and K is a constant used to fit the model to the data set.

For the Finite Element Analysis (FEA), a three-dimensional model was created in Ansys Spaceclaim software (ANSYS, Inc.; Canonsburg, PA, USA). The model consisted of 3 layers, following the same configuration of the in vitro test: piston, ceramic, and dentin analog substrate. The cement layer was neglected. The model was imported into Ansys 19.0 software (ANSYS, Inc.; Canonsburg, PA, USA).

The FFC group consisted of cylindrical geometry (12 mm in diameter and 33 mm in length) with a conical end of 6 mm in length and a flat point of 3 mm in diameter. The SFC group consisted of cylindrical geometry (5.72 mm in diameter and 58.40 mm in length). The base consisted of a cylinder 4 mm thick and 20 mm in diameter. The mesh was composed of hexahedral parabolic elements. The FFC model was constituted by 43936 elements and 185075 nodes; while the SFC model had 35577 elements and 153780 nodes. Both models had a 0.3 mm element size. Mesh refinement was based on the convergence of stresses at the interface between ceramic and G10 dentin analog. 

The contact between the piston and the ceramic was defined as frictional contact (friction coefficient=0.2) and between the ceramic and the G10 substrate as bonded contact. The lower side of the G10 substrate was fixed in all translations. In the upper surface of the piston was applied a compressive load of 30 N and 80 N, and the piston was configured for moving only in the load direction.

The with material’s elastic properties (elastic modulus and Poisson's ratio) were attributed to the respective layers of the model (Table 1) ^18^ ^,^ ^25^. The materials were considered homogeneous, isotropic, and with linear elastic behavior. The results analysis was performed based on the location and values of maximum principal stresses (MPa), considering that fracture of brittle materials is mainly attributed to the presence of tensile stresses.

**Table 1:** Values of elastic modulus (E) and Poisson's ratio (() of materials used in the static simulation.

Materials	E (GPa)	(
Glass-fiber reinforced epoxy resin - G10^*^	14.9	0.31
Leucite-reinforced glass-ceramic^25^	65	0.25
Stainless steel ^22^	200	0.30

* Materials library (Solidworks Corp).

## Results

For the monotonic compressive load test, there was no difference between the groups for L_0_ and *m*, since the confidence intervals overlapped (Table 2).

**Table 2:** Characteristic fracture load (L_0_) and Weibull modulus (*m*) values for specimens tested in monotonic compressive load using flat-to-flat (FFC) and spherical-to-flat (SFC) contact conditions, and their respective 95% confidence intervals (95% CI). Values were considered statistically similar when the 95% CI overlapped.

Group	L_0_ (N)	95% CI - L_0_ (N)	*m*	**95% CI - *m***
FFC	1111^a^	1034; 1196	6^a^	4;8
SFC	1037^a^	951; 1133	5^a^	3;6

*Values followed by similar letters in the same column are statistically similar.

The fatigue parameters for SFC were β = 0.95, K = 1.9×10^-13^, *n* = 4.00; and for FFC were β = 0.86, K = 3.86×10^-7^, *n* = 0.74. The fatigue Weibull modulus (() was similar among groups. The parameter *n* is the slow crack growth exponent, which measures the effect of the load level on the specimens P_f_. A greater *n-*value (*n*>1), as observed for SFC, indicates a greater effect. When the *n-*value approaches zero, as seen for FFC, there is only a small effect of the load on the P_f_ of specimens tested in fatigue.

Table 3 shows the P_f_ predictions^*^ at 30 N to 80 N loads (values in the range of intraoral loads)^5^ ^,^ ^9^ and 200,000 cycles. P_f_ predictions were based on the statistical model used to analyze the fatigue data. These different load levels were chosen aiming to show the load-dependency of the P_f_ for SFC; while for FFC, the P_f_ was mostly constant over the different load levels.

**Table 3:** Probability of failure (P_f_) estimated at 30 N to 80 N loads (values in the range of intraoral loads) for 200,000 cycles, with respective 95% confidence intervals (95% CI).

	Groups			
	SFC		FFC	
Load	P_f_ (%)	95% CI	P_f_ (%)	95% CI
30 N	4	0; 33	62	36; 87
40 N	11	2; 45	67	45; 89
50 N	23	8; 57	74	44; 95
60 N	41	22; 68	78	40; 99
80 N	79	68; 89	83	32; 99

**Figure 1:**
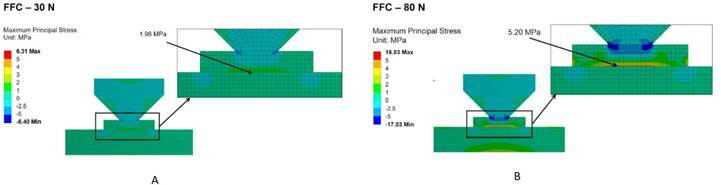
Cross-sections of the FEA models simulating flat-to-flat contact (FFC) showing the maximum principal stress distribution of specimens tested under 30 N (A) and 80 N (B) compressive load.

**Figure 2:**
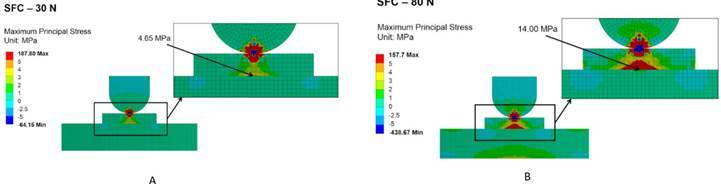
Cross-sections of the FEA models simulating sphere-to-flat contact (SFC) showing the maximum principal stress distribution of specimens tested under 30 N (A) and 80 N (B) compressive load.

Figures 1 and 2 show the maximum principal stress distribution of specimens for SFC and FFC conditions under 30 N and 80 N compressive load as to characterize the behavior of the materials at a low and high load levels. For FFC it is possible to observe compressive stresses at the piston contact surrounding area and tensile stresses at the interface between ceramic and substrate (Fig. 1). When the compressive load increases from 30 N to 80 N, it is possible to observe an increase in the area of the specimen subjected to tensile stresses (intaglio surface) followed by a small increment in the values of maximum principal stress and homogeneous stress distribution.

For SFC, both tensile and compressive stresses are located at the contact and near-contact areas, while tensile stresses are present at the interface between ceramic and substrate (Figure 2). A wider area of the ceramic specimen is subjected to the high concentration of tensile stresses in both contact and intaglio surfaces when the load increases from 30 N to 80 N.

**Figure 3:**
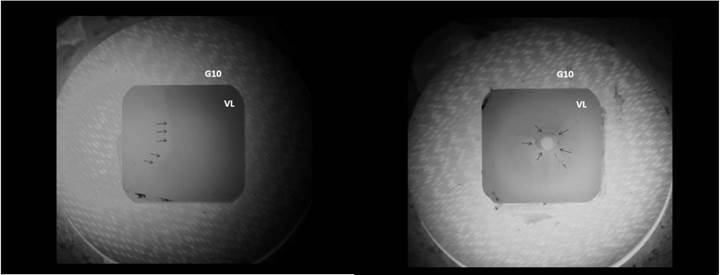
Representative images of the failure modes observed for the experimental groups. (A) Arrows point to a radial crack found in a specimen tested in fatigue. (B) Arrows point to a cone crack observed in a specimen tested in monotonic compressive load. VL - leucite-reinforced glass-ceramic. G10 - dentin analogue substrate.

Chipping and catastrophic failures were not observed for the evaluated ceramic specimens. Cracking was the predominant failure mode, being the radial crack (Figure 3) the most frequent type of crack (Table 4)

**Table 4:** Frequency of each type of crack for specimens tested in flat-to-flat (FFC) and spherical-to-flat (SFC) contact conditions using monotonic and cyclic fatigue tests.

Monotonic Compressive Load Test							
Group		n	Radial	Cone	Combined		
FFC		20	17 (85%)	3 (15%)	0 (0%)		
SFC		20	12 (60%)	4 (20%)	4 (20%)		
Cyclic Fatigue Test							
Group	N. cycles	Load	n	Radial	Cone	Combined	Survived
FFC	100,000	69 N	10	10 (100%)	0 (0%)	0 (0%)	0 (0%)
	100,000 ? 200,000*	56 N	10	7 (70%)	0 (0%)	0 (0%)	3 (30%)
	200,000	49 N	10	7 (70%)	0 (0%)	0 (0%)	3 (30%)
SFC	100,000	98 N	10	8 (80%)	1 (10%)	1 (10%)	0 (0%)
	100,000 ? 200,000*	81 N	10	3 (30%)	1 (10%)	4 (40%)	2 (20%)
	200,000	69 N	10	6 (60%)	0 (0%)	0 (0%)	4 (40%)

* For this protocol, specimens that survived the first lifetime were allowed to run out through the second lifetime and no specimens failed between 100,000 and 200,000 cycles, so the failure rate remained the same. Cracks described in the Table were observed after 100,000 cycles.

## Discussion

The loading conditions affected the mechanical behavior and stress distribution of the bonded leucite-reinforced glass-ceramic, accepting the study hypothesis. The fatigue parameter *n* (slow crack growth exponent), which measures the effect of the load level on the specimen’s probability of failure, was greater for SFC. The load-dependency was also evident when the probability of failure predictions were made at different load levels, and same number of cycles, for the two contact conditions (Table 3). The estimated P_f_ for specimens tested with SFC increased from 4% to 79% as the load increased from 30 N to 80 N; while for FFC the P_f_ was mostly constant, varying from 62% to 83%.

When a compressive load is applied to a ceramic layer cemented to a compliant substrate, localized stresses are created in the loading contact area (compressive) and at the near-contact regions (tensile) ^4^ ^,^ ^5^ ^,^ ^10^ ^,^ ^17^. The elastic properties of the substrate (G10, E=15 GPa) ^18^ induce the ceramic (leucite-reinforce glass-ceramic, E=65 GPa) ^20^ deflection under loading, resulting in tensile stresses concentration at the intaglio surface ^4^ ^,^ ^5^ ^,^ ^12^ ^,^ ^14^. For SFC, a bell-shaped zone of tensile stresses is located in the intaglio surface; while for FFC, this zone has a different geometry and the surface area in tension is wider. Nevertheless, changes in the specimens stress distribution when FEA was performed at different load levels were more abrupt for SFC, which could explain the greater effect of load in the failure behavior.

The tensile stresses located in the ceramic intaglio surface could initiate radial cracks, which was the most frequent failure mode for all testing conditions. Nevertheless, both tensile and compressive stresses are located at the contact and near-contact areas of specimens tested in SFC. The contact radii of the spherical piston with the ceramic flat surface varies according to the load level applied due the deformations of materials ^6^ ^,^ ^10^. For high compressive loads, the contact radii and pressure increase, which could modify the failure mode as well. Studies have showed that radial and cone cracks are competing failure modes ^4^ ^,^ ^5^ ^,^ ^14^. The dominant failure mode for thin glass-ceramic specimens tested in cyclic contact fatigue with low load levels is radial crack ^5^, as observed in the present study. Yet, higher loads can increase the magnitude of stresses at the loading contact surrounding area, which could contribute to cone cracks formation ^5^. In addition, a higher number of cycles can accumulate damage at the contact surface ^17^. In the present study, for SFC, cone cracks (combined or not with radial cracks) were present when specimens were cycled at loads above 80 N. Clinically, both radial and cone cracks can be found ^2^ ^,^ ^6^.

In fatigue, when we compare the P_f_ estimated for lower loads (30 N and 40 N) and 200,000 cycles, specimens tested with the SFC condition showed lower P_f_ than the ones tested with the FFC condition. Nevertheless, the groups showed similar P_f_ when estimations were performed at higher loads (50 N to 80 N). These findings are related to the differences in the fatigue behavior (*n*-value), and corroborate with the monotonic compressive load test results, in which high values of fracture load were recorded (above 800 N) and there were no differences among groups. Moreover, there were no differences for the monotonic (*m*) and the fatigue (() Weibull modulus among groups, which suggests a similar flaw size distribution and results in similar variability in the probability of failure estimations ^21^.

The literature recommends using flat-to-flat loading conditions to guarantee a constant and uniform pressure in order to simulate the contact facets between the opposing teeth and to avoid contact damage ^3^ ^,^ ^6^. The study findings showed only a small effect of the load level on the stress distribution and probability failure for specimens tested in fatigue using FFC, which is an advantage that should be considered when designing a mechanical test. However, the contact area of flat pistons is limited by a sharp edge at the borders, which could induce high stress levels to the ceramic top surface, as shown in the FEA, meaning that this piston may not be adequate when high loads are used in the test. Therefore, two different approaches can be used for laboratory testing with hard pistons. The first approach is to use the metallic piston in a sphere-to-flat condition; taking into account the load-dependence previously discussed. The other option is to use the flat metallic piston when fatigue tests are performed with low loads, to avoid the contact damage from the sharp edges. Hard pistons have the advantage of preserving their integrity during the experiments. Yet, a soft material can also be recommended to test ceramic specimens in a flat-to-flat loading condition ^3^ ^,^ ^6^ ^-^ ^9^ ^,^ ^13^. A previous study showed longer lifetimes and lower probability of failure for bonded leucite-reinforced glass ceramic tested in fatigue using a flat piston produced with glass-fiber reinforced epoxy resin composite ^7^.

Fatigue testing of bonded flat ceramic specimens aim to create the same crack system as seen in bulk clinical failures, ^6^ ^,^ ^16^ ^)^ but neglect the effect of the restoration geometry in the stress distribution, which is a study limitation. In addition, although the parameters used in the fatigue test were chosen considering intra-oral variables, ^3^ ^,^ ^4^ ^,^ ^6^ ^,^ ^8^ ^,^ ^12^ ^,^ ^16^ the specimens high failure rate did not allow for evaluating its fatigue behavior at longer lifetimes. Moreover, it is important to emphasize that a linear finite element analysis was used as an additional tool to understand the stress distribution at the ceramic intaglio surface, considering that radial cracks originated in this area were the most frequent failure mode. Yet, the finite element model considers a perfect union (free of defects) between the layers, which could lead to an underestimation of the stress magnitude at the intaglio surface. In addition, the cement layer was not included in the model due to its small thickness (~50 µm). A more complex analysis would be required to simulate the non-linear contact behavior of the spherical piston with the ceramic surface and the effect of the cement layer, which is a recommendation for future studies.

It can be concluded that the loading conditions affected the stress distribution and mechanical behavior of a leucite-reinforced glass-ceramic bonded to a dentin analog. The stress distribution and probability of failure of specimens tested with sphere-to-flat contact conditions showed greater dependency to load level.
